# Acute Myeloid Leukemia With a Non-Canonical *FLT3* V491L Mutation: A Case Report With *Ex Vivo* FLT3 Inhibitors Sensitivity Testing

**DOI:** 10.14740/jmc5197

**Published:** 2025-12-24

**Authors:** Mateusz Pospiech, Michelle P. Ngo, Misk Alrawashdeh, Usama Qamar, Amir Ali, Eric Tam, George Yaghmour, Houda Alachkar, Abdullah Ladha

**Affiliations:** a Department of Clinical Pharmacy, Alfred E. Mann School of Pharmacy and Pharmaceutical Sciences, University of Southern California, Los Angeles, CA 90089, USA; b Jane Anne Nohl Division of Hematology and Center for the Study of Blood Diseases, Norris Comprehensive Cancer Center, Los Angeles, CA, USA; c Department of Medicine, Aga Khan University, Karachi, Pakistan; d Honor Health Cancer Transplant Institute, Scottsdale, AZ, USA

**Keywords:** Acute myeloid leukemia, Gilteritinib, *FLT3* V491L mutation

## Abstract

Approximately 30% of patients with acute myeloid leukemia (AML) harbor FMS-like tyrosine kinase 3 (*FLT3*) mutations, which are associated with poor overall survival. Although United States Food and Drug Administration (FDA)-approved FLT3 inhibitors are available, their efficacy against non-canonical *FLT3* mutations remains elusive. Here we present a case of a 72-year-old female Jehovah’s Witness with newly diagnosed AML carrying a rare pathogenic *FLT3* V491L mutation identified by next-generation sequencing. Given the patient’s religious beliefs, blood transfusion was not an option, making the patient ineligible for high-intensity chemotherapy and leading to alternative treatment approaches. To our knowledge, this is the first case report of the effectiveness of gilteritinib in an older patient with AML with a non-canonical *FLT3* mutation and limitation on blood products usage. Initial treatment with hydroxyurea and leukapheresis followed by azacitidine and venetoclax resulted in an inadequate treatment response. Given the lack of research on the *FLT3* V491L mutation, we conducted an *ex vivo* sensitivity study using the patient’s diagnostic bone marrow blasts to assess and compare the anti-leukemic efficacy of midostaurin, quizartinib, and gilteritinib. The *ex vivo* study revealed the lowest half-maximal inhibitory concentration (IC_50_) value and the highest number of apoptotic cells in gilteritinib treated patient’s blasts under Flt3 ligand-supplemented conditions. An initial clinical improvement with gilteritinib was observed. However, after the third cycle, gilteritinib was substituted with midostaurin because of high copay costs with gilteritinib. Subsequently, an increase in leukemic blasts was observed, and soon after, the patient expired. Treatment of relapsed AML with a non-canonical mutation is challenging due to the lack of data regarding FLT3 inhibitors. This case highlights the potential role of gilteritinib in targeting the rare *FLT3* V491L mutation, underscoring the need for further research and improved accessibility to effective therapies.

## Introduction

FMS-like tyrosine kinase 3 (*FLT3*) mutation is a common prognostic and predictive marker, found in approximately 30% of patients with acute myeloid leukemia (AML) [1, 2]. It is classified as an internal tandem duplication (ITD) or, less often, as a tyrosine kinase domain (TKD) mutation in 25% and 7-10% of patients, respectively [2]. FLT3 activation leads to downstream leukemic cell proliferation. Various ITD and TKD mutations have been reported at the time of diagnosis, which can predict sensitivity to different FLT3 inhibitors (FLT3i) [3]. Non-canonical (NC) mutations comprise 21% of *FLT3* mutations. *FLT3* V491L is a rare NC *FLT3* mutation; it is classified as a non-recurrent point mutation of extracellular immunoglobulin-like domain [4]. Tarver et al showed that gilteritinib was effective in various NC mutations and was less vulnerable to resistance [5]; however, evidence regarding the efficacy of FLT3i in this rare mutation is currently lacking. Here, we describe a clinical case and an ex vivo study to investigate the effect of FLT3i on leukemic blast cells with *FLT3* V491L mutation. As gilteritinib has broader activity against *FLT3* mutations, we hypothesized that it would show better efficacy against *FLT3* V491L mutation than other FLT3i. Therefore, we conducted ex vivo drug sensitivity testing to evaluate the anti-leukemic effects of midostaurin, quizartinib, and gilteritinib on the patient’s diagnostic bone marrow blasts.

## Case Report

### Investigations and diagnosis

A 72-year-old female patient presented with newly diagnosed AML based on peripheral blood flow cytometry, which showed 40-45% blasts on the peripheral smear at the time of diagnosis in February 2024. Peripheral blood smear revealed moderate macrocytic anemia, and moderate thrombocytopenia along with the presence of leukemic blasts. Diagnosis of AML was confirmed by bone marrow biopsy. Ancillary testing results showed AML with partial monocytic differentiation (95% of total events). Immunophenotyping showed that myeloid blasts (80%) were positive for CD34 (partial), CD117, human leukocyte antigen-DR (HLA-DR), CD33, CD13 (subset), CD64 (dim/partial), CD 11c (partial), and myeloperoxidase (MPO), and negative for terminal deoxynucleotidyl transferase (TdT), B-cell and T-cell markers. Atypical immature monocytes (15%) were positive for CD4 (dim), CD11c, CD14 (partial), CD33 and CD64, and negative for CD16. Next-generation sequencing (NGS) revealed a myelodysplasia-related *SRSF2* mutation (p.P95H, variant allele frequency (VAF) 47.3%) and pathogenic mutations in *FLT3* V491L (VAF: 40.3%), *CSF3R* T618I (VAF: 5.5%), and *TET2* (VAF p.Q1627Hfs*63 48.1%; p.H1380N 46.9%). A prior report showed that co-mutation of *FLT3*-ITD and *TET2* could suggest that combined targeting of FLT3 signaling and epigenetic pathways could increase response to treatment [6].

### Treatment and outcome

**Figure 1:**
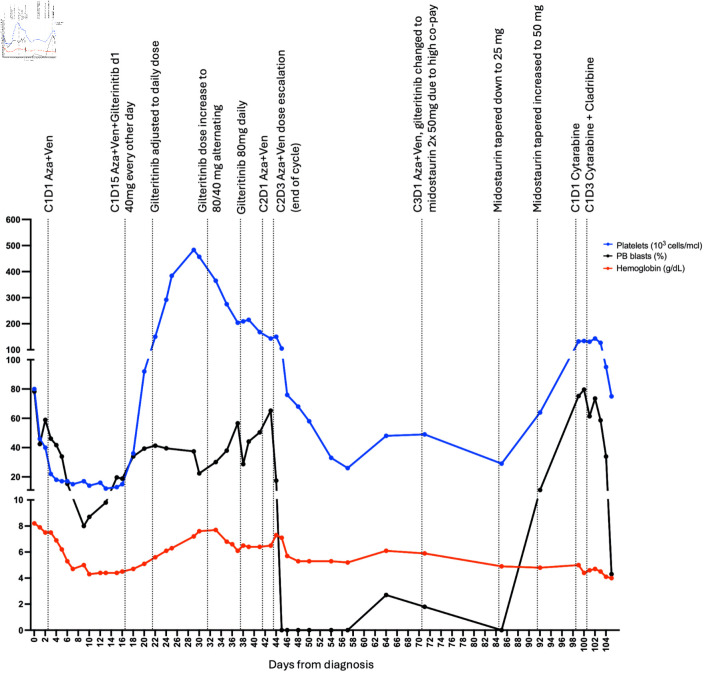
Disease treatment and progression. Each point represents measurement of peripheral blood (PB) blast percentage (black), hemoglobin level (red) or platelets measurement (blue). First point represents diagnosis at day 0, C1D1 Aza + Ven represents cycle one day 1 of the treatment with azacitidine (Aza) and venetoclax (Ven), C1D1 cytarabine indicates cycle one day 1 of cytarabine treatment, C1D3 cytarabine + cladribine indicates cycle one day 3 of cytarabine and cladribine treatment. Last point represents the last day at the hospital, soon after the patient expired at a different institution.

The standard of care would suggest initiating the induction (7 + 3) protocol for newly diagnosed AML patients. However, since the patient identifies as a Jehovah Witness, administration of blood products such as whole blood, packed red blood cells, white blood cells, plasma and platelets including autologous transfusions is excluded from treatment options. The limited possibility of blood transfusions to manage chemotherapy-induced bone marrow suppression and associated complications, such as anemia or thrombocytopenia, led to alternative treatment approaches. Upon admission to the cancer center, the patient received hydroxyurea treatment (2 g) followed by leukapheresis for leukocytosis (white blood cells: about 100 × 10^3^/µL) before receiving reduced doses of azacitidine (Aza, 50 mg/m^2^) and venetoclax (Ven, 50 mg) [7] to accommodate these restrictions while providing effective treatment. On days 1 - 3, she was given Aza 50 mg/m^2^ and Ven 50 mg, and Aza 25 mg/m^2^ on day 4. Aza was held on day 5, given worsening hemoglobin levels (6.9 g/dL). Despite stable pancytopenia, blasts increased from 9.8% on day 13 to 19.6% on day 15. The presence of an atypical *FLT3* mutation prompted the use of gilteritinib (cycle one, day 15) at 40 mg every other day, a FLT3i that is approved by the US Food and Drug Administration (FDA) as a single therapeutic agent in relapsed or refractory AML [8]. While the standard therapeutic dose of gilteritinib is 120 mg daily, given low hemoglobin (4.5 g/dL) and platelets levels (15 × 10^3^/µL), a reduced dose of 40 mg gilteritinib every other day was started on day 15. On day 20, gilteritinib was titrated up to 40 mg once daily for 10 days, and then (cycle one, day 30) the dose was alternated between 40 mg and 80 mg every other day for 1 week. The patient tolerated the dose well and showed a response to treatment (Fig. 1).

The dose was further increased to 80 mg once daily (cycle one, day 36). Cycle two of azacitidine and venetoclax started with: Aza 50 mg/m^2^ and Ven 50 mg for days 1 - 2 and increased to Aza 75 mg/m^2^ and Ven 100 mg for day 3. Due to down trending platelets (cycle two, day 1, 168 × 10^3^/µL to 143 × 10^3^/µL on day 3) and low hemoglobin (6.5 g/dL), treatment ended after day 3. Aza and Ven were limited to 3 days, but gilteritinib 80 mg daily was continued, and peripheral blasts continued to be not detected. A month later, cycle three proceeded with 3 days of Aza 50 mg/m^2^ and dose-reduced 100 mg venetoclax. Another FLT3i, midostaurin 50 mg twice daily, replaced gilteritinib due to high copay and insurance issues. At a follow-up visit, midostaurin was tapered down to 25 mg once daily as no peripheral blasts were identified and to avoid myelosuppression. However, the dose was increased back to 50 mg twice daily a week later after an increase in blasts (11%).

Within a few weeks, the patient developed hyperleukocytosis, which was treated with cytarabine 20 mg/m^2^ every 12 h on days 1 - 8, cladribine 2.5 mg/m^2^ on days 3 - 7, and low-dose venetoclax. Unfortunately, the patient suffered septic shock and passed away with refractory disease.

### Ex vivo sensitivity testing

#### Ex vivo sensitivity testing methods

##### 1) Cell culture

Bone marrow cells with approximately 85% leukemic blasts were retrieved from the leukemia biorepository bank at the USC Norris Cancer Center and cultured overnight in RPMI1640 with 20% fetal bovine serum (FBS) (R20), supplemented with CC100 (02690, StemCell Technologies) cytokine cocktail containing interleukin 3, interleukin 6, stem cell factor, and Flt3 ligand. The following day, cells were counted using trypan blue (15250061, ThermoFisher) before the experimental setup.

##### 2) Viability assay

Approximately 50,000 cells/well in 50 µL of R20 supplemented with CC100 were seeded into a 96-well plate (25-104, Genesee). FLT3 inhibitor (2 ×) solutions were prepared in R20 with concentrations ranging from 0.0037 µM to 1 µM and added to seeded cells in triplicates. Cells were incubated for 72 h before the addition of 10 µL of cell counting kit-8 (CCK-8) reagent (ab228554, Abcam). After 4 h of incubation, absorbance at 460 nm was measured on a Synergy H1 plate reader. The half-maximal inhibitory concentration (IC_50_) values were obtained using dose-response curve fitting in GraphPad Prism v10.1.1.

##### 3) Cell killing assay

Approximately 500,000 cells/well in 500 µL of R20 supplemented with CC100 were seeded into a 12-well plate. FLT3 inhibitor (2 ×) solutions were prepared in R20 at final concentrations of 1 µM and 0.5 µM and added to the seeded cells in triplicates. Cells were incubated for 72 h. Then, 10 µL of the cell suspension was mixed with 10 µL of trypan blue (15250061, ThermoFisher) and counted using a hemacytometer; cells in each well were counted three times. The remaining cell suspension was used to evaluate cell death by staining with propidium iodide (PI). Samples were run on a Fortessa X20 Flow cytometer and analyzed using FlowJo v 10.8.1.

#### Ex vivo sensitivity testing results

**Figure 2:**
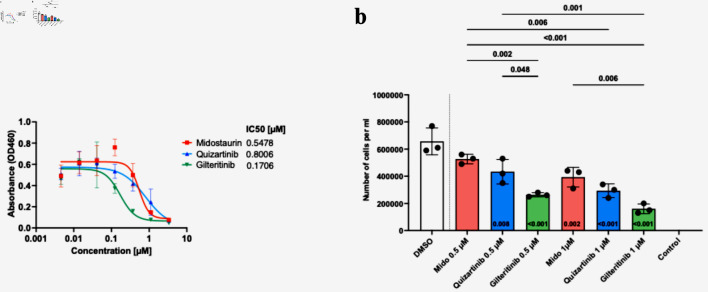
Patient blasts show variable responses to FLT3 inhibitor treatment, with the highest efficacy of gilteritinib. Cells were seeded with graded concentrations (0 - 3.33 µM) of midostaurin, quizartinib and gilteritinib for 72 h, and cell viability was determined by CCK-8 assay. The graph depicts the IC_50_ values for each drug (a). Cells were seeded with 0, 0.5, or 1 µM of FLT3 inhibitor or dimethyl sulfoxide (DMSO). Cells were counted three times 72 h post-treatment with trypan blue (b). Statistical analysis was performed using one-way ANOVA, with adjusted P < 0.05 considered significant.

**Figure 3:**
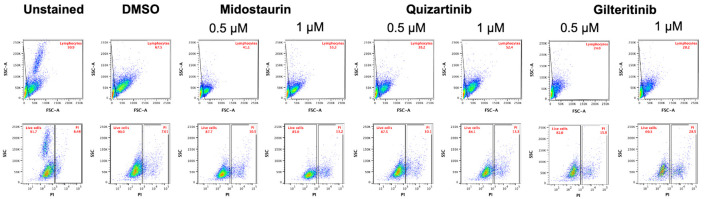
Gilteritinib treatment results in the lowest percentage of live cells among tested FLT3 inhibitors. Flow cytometry analysis of cells stained with propidium iodide (PI) was performed to evaluate cell death. Cells were first gated for the lymphocyte population based on side scatter and forward scatter, followed by gating cells on PI-positive and PI-negative (live cells) populations in reference to unstained control. FLT3: FMS-like tyrosine kinase 3; DMSO: dimethyl sulfoxide; FSC-A: forward scatter area.

Viability assay using CCK-8 assay showed that the patient’s blasts exhibited variable sensitivity to the FLT3i (midostaurin IC_50_ = 547.8 nM; quizartinib IC_50_ = 800.6 nM; gilteritinib IC_50_ = 170.6 nM) (Fig. 2a). Similarly, a significant decrease in the number of live cells at 72 h was observed following cell’s incubation with 1 µM of FLT3i (Fig. 2b). Gilteritinib treatment showed significantly higher effectiveness compared with midostaurin at both 0.5 and 1 µM treatment. Similarly, 0.5 µM gilteritinib was more effective in decreasing cell numbers compared with 0.5 µM quizartinib (Fig. 2b). Lastly, cell death measured by flow cytometry showed the lowest number of live cells in the 1 µM gilteritinib treatment (Fig. 3).

The study was approved by the University of Southern California Institutional Review Board and conducted in accordance with the ethical standards of the 1964 Declaration of Helsinki and its later amendments or comparable ethical guidelines.

## Discussion

To date, there has been little research done on *FLT3* V491L mutation. It has been identified by sequencing but has not been characterized [4, 9, 10]. It is a rare NC *FLT3* mutation within the immunoglobulin (Ig)-like domain potentially affecting ligand binding and protein function (ligand binding domain) of *FLT3* [4]. FDA-approved treatments for *FLT3* mutations include midostaurin (a first-generation inhibitor for *FLT3*-TKD and *FLT3*-ITD mutations) combined with chemotherapy [11]; gilteritinib (a second-generation inhibitor) as a single agent for relapsed or refractory AML [8]; and quizartinib (another second-generation inhibitor) in combination with chemotherapy for newly diagnosed *FLT3*-ITD AML patients [12]. Quizartinib, gilteritinib, and midostaurin act on *FLT3* mutations, but midostaurin has off-target effects related to inhibition of other tyrosine kinase receptors as well [13, 14]. Quizartinib is the only approved FLT3i that is designed to target only ITD mutation, whereas midostaurin and gilteritinib also target TKD mutation [8, 11, 12, 15].

Tarver et al showed that gilteritinib was effective in various NC mutations and was less vulnerable to resistance [5]. Ge et al found that V491L mutation was only present in 0.3% and 1% of AML and AML with *FLT3* mutation, respectively [4]. Ge et al [4] classified V491L as non-recurrent point mutation, which collectively had improved outcomes compared to *FLT3* TKD. Gilteritinib has also been shown to work against other NC *FLT3* mutations [5]. Despite the lower therapeutic dosing of gilteritinib, the observed therapeutic response highlights the improved activity of gilteritinib. Managing AML patients with transfusion limitations is a clinically dynamic process. In our case, doses of azacitidine and venetoclax were significantly curtailed, but we found a meaningful clinical response, including stability in cytopenia and disappearance of peripheral blasts, when the patient was able to maintain lower therapeutic doses of gilteritinib. These clinical findings align with *ex vivo* sensitivity data for the V491L *FLT3* mutation.

Several mechanisms can explain FLT3i resistance. Elevated levels of FLT3 ligand have also been observed in relapse patients who previously received FLT3i treatment during their first induction therapy [16], which could lead to midostaurin resistance after the rise in blasts in this case. Another possibility is a change of clonal dominance at relapse from FLT3 to CSF3R clones, which are JAK-STAT dependent, possibly giving rise to FLT3i resistance [17]. Acquisition of additional mutations during the relapse in the TKD domain could also lead to increase in AXL levels [18], which was previously shown to contribute to midostaurin and quizartinib resistance [18, 19]. As it is one of the off-targets of gilteritinib, this may possibly explain the efficacy *in vivo* [20].

The use of NGS-based mutation testing is standard in AML. It frequently detects NC mutations, which are not well known, such as *FLT3* V491L. Our case study and *ex vivo* data present improved efficacy of gilteritinib compared to midostaurin and quizartinib in treating AML with NC *FLT3* V491L mutation. However, previous studies showed that FLT3 ligand may preferentially inhibit midostaurin and quizartinib function while having little impact of inhibitory properties of gilteritinib [16, 21], which could possibly introduce bias in the *ex vivo* study.

Limited studies have highlighted the role of specific FLT3i in NC *FLT3* mutations. Few studies reported IC_50_ values for some individuals carrying V491L mutation [22, 23]. A study by Tyner et al reported a patient with the *FLT3* V491L mutation whose blasts were screened for sensitivity to midostaurin and quizartinib, yielding IC_50_ values of 0.857 µM and 14 nM, respectively, indicating higher sensitivity to quizartinib [22]. In the study by Bottomly et al, one patient with 3% VAF of *FLT3* V491L exhibited IC_50_ values of 10 µM (non-responder) for midostaurin, 52 nM for quizartinib, and 14 nM for gilteritinib [23]. Another patient with 53% VAF of *FLT3* V491L mutation showed IC_50_ values of 2.59 µM for midostaurin, 167 nM for quizartinib and 58 nM for gilteritinib. Similarly, a third patient with 51% VAF of V491L mutation was reported to have IC_50_ values of 0.855 µM for midostaurin, and 14 nM for quizartinib, while the blasts were not tested for response to gilteritinib. While those studies indicated the highest efficacy with gilteritinib, we also reported higher IC_50_ values for both quizartinib and gilteritinib than those previously reported, possibly due to the use of *FLT3* ligand in our experimental setting, which may limit the comparative value between type I and type II FLT3i. However, better efficacy of gilteritinib treatment in this patient’s blasts is consistent with previous findings. Our findings support using gilteritinib for the rare NC V491L mutation in this case, although the rarity of V491L *FLT3* mutation makes generalizability of our findings limited. Our results highlight the need for a more personalized approach for the treatment of patients with rare NC mutations and drug testing to ensure better therapies can be utilized to control disease. It is also important to note the impact of financial barriers in this case, where despite the clinical efficacy of gilteritinib, high copay costs led to a switch to midostaurin. This access limitation underscores the influence of financial constraints on therapeutic decisions. In clinical practice, it can be easy to disregard NC mutations because they are rare, and their therapeutic implications are unknown. Although our study has limitations, we present a systematic approach to managing patients with these mutations. This becomes especially important when traditional myelosuppressive chemotherapeutic agents cannot be used, such as in Jehovah’s Witness patients.

### Learning points

Jehovah’s Witness patients with AML are not ideal candidates for intensive chemotherapy due to transfusion restriction and present a significant clinical challenge, which needs innovative solutions in future studies.

In AML cases with rare NC mutations, individualized ex vivo drug sensitivity testing can potentially help with treatment planning.

Gilteritinib is an effective therapy for rare NC *FLT3* V491L mutation as shown with case presentation and ex vivo drug sensitivity; these results should be confirmed in future studies.

## Data Availability

The data supporting the findings of this study are available from the corresponding author upon reasonable request.
